# Incidental finding of prostate cancer in Transurethral Resection of Prostate (TURP) specimens: a retrospective analysis from a Tertiary Care Hospital in Pakistan

**DOI:** 10.11604/pamj.2021.39.20.26931

**Published:** 2021-05-07

**Authors:** Taimoor Khalid Janjua, Muhammad Ali Yousuf, Muhammad Talha Iqbal, Shahbaz Mustafa Memon, Aziz Abdullah, Naveen Faridi, Muhammad Irfan

**Affiliations:** 1 Liaquat National Medical College, Liaquat National Hospital, Karachi, Pakistan,; 2 Urology Department, Liaquat National Hospital, Karachi, Pakistan,; 3 Surgery Department, Sherwood Forest Hospitals, NHS Trust, Nottinghamshire, United Kingdom,; 4 Pathology Department, Liaquat National Hospital, Pakistan,; 5 Biostatistics Department, Liaquat National Hospital, Karachi, Pakistan

**Keywords:** Incidental prostate cancer, TURP specimens, Pakistan

## Abstract

**Introduction:**

incidental prostate cancer findings reflect the great burden of prostatic cancer across the globe. Our 10 year retrospective analysis aimed to identify the incidence and clinic-pathologic features of prostate cancer incidentally detected in patients undergoing transurethral resection of the prostate (TURP) for benign prostatic hyperplasia (BPH), and to estimate the clinical value of pathologic review of all TURP specimens.

**Methods:**

after excluding patients with a known diagnosis of prostate cancer prior to TURP a total of 2,386 men (ages 25-98) were identified by pathology (TURP) specimens. Yearly incidences, Gleason score, grade, pathologic stage were recorded for all incidental prostate cancer patients.

**Results:**

a total of 256 (10.7%) patients were found to have prostate cancer. Mean Age was 68.51±9.22 years. T1a and T1b stage prostatic carcinoma was found in 9.9% and 90.1% of these patients respectively. Forty-nine percent (49%) patients had higher Gleason scores (>7). After subtracting average incidences between 5-year intervals, a statistical rise of almost 4% was found.

**Conclusion:**

our analysis concludes that a large proportion (10.7%) of patients had incidental prostate cancer and the incidence was increasing in recent years in Pakistan and in comparison, to Asian countries. In Pakistan there is a scarcity of updated national cancer registries. The growing incidence of high Gleason scores requires keen and prompt attention. The diverse ethnic and socioeconomic background of patients propels their propensity towards loss of follow up with already limited tertiary healthcare institutes in Pakistan. This pathologic review of TURP specimens is valuable for Asiatic and non-Asiatic populations.

## Introduction

Prostate cancer is the second most common cancer in men worldwide and according to recent data over 1.3 million cases of prostate cancer were reported worldwide [1]. Developing countries express a lower prostatic cancer incidence when juxtaposed with developed nations [2]. However the incidence of prostate cancer in Pakistan is increasing [3]. An observation of approximately 60% increase in Prostate Cancer was concluded from 1995 to 2002 [4]. Age is one of the most commonly associated risk factors for development of prostate cancer [5]. Prostate Cancer is diagnosed and differentiated from another pathology, Benign Prostatic Hyperplasia (BPH) through obtaining biopsy and performing histopathological analysis which is the Gold standard method [6]. The Gleason grading and scoring criteria is internationally and commonly utilized for grading and scoring prostate adenocarcinomas [7]. Transurethral Resection of Prostate (TURP) and prostatectomy are commonly implied methods for obtaining Prostate tissue for histopathological diagnosis of benign conditions of the prostate gland like Benign Prostatic Hyperplasia [8-10]. Statistics from an Indian study revealed other premalignant conditions as well and interesting associations of Benign Prostatic Hyperplasia (BPH), prostatic adenocarcinoma and Gleason scoring system with age groups [11]. A Chinese population study involving prostate cancer reporting through TURP specimens revealed increasing trend [12]. Studies investigating Prostatic Cancer concluded positive associations of higher age groups (above and equal 65) with higher tumor incidence where histopathological specimens were obtained through TURP [13,14]. TURP mainly targets the transitional zone of prostate Gland where about 25% of tumors are found. Majority are found in the peripheral zone [15]. In our hospital setting patients are commonly and increasingly presenting with Benign Prostatic Hyperplasia (BPH) and incidental prostate cancer in TURP specimens.

## Methods

This 10 year retrospective analysis of TURP specimens collected between from January 2007 and before January 2017 was conducted at the Histopathology Department of the Liaquat National Hospital and Medical College in Karachi, Pakistan. TURP specimens were obtained by urologists of the hospital and histopathology reports were prepared and finalized by two pathologists of the Histopathology department. Patients of all age presenting to the Urology clinic requiring TURP procedures were included. Patients with known or existing prostate cancers, other primary and secondary carcinomas were excluded. Respective approvals for this study were obtained from the research and ethics committee of the institute.

**Data analysis procedure**: SPSS 22(Chicago, Illinois) was used for data collection and analysis.

## Results

The mean age of all patients who underwent TURP and having incidental prostate cancer patients was 68.51with a standard deviation (SD) of 9.22 years respectively. As illustrated in Table 1 the 6^th^ and 7^th^ decades of life were the most common for all patients presenting with incidental prostatic carcinomas. The incidence of prostate cancer among all 2386 TURP specimens was calculated as 10.72%. Majority patients (90.9%) with incidental carcinoma were pathologically staged T1b. A difference between averages of two 5 year intervals i.e. [2007-2011] and [2012-2016] revealed a 3.92% difference i.e. an approximate numerical rise of 4%. Majority patients (49.21%) had higher (>7) Gleason scores.

**Table 1:** year-wise quantitative analysis

Year	Total Biopsy Specimens (n)	Most common, 2^nd^ most common decade for Prostatic Cancer specimens	Prostate Cancer Incidence%	High Gleason Score(>7) %
	[BPH + Prostatic Cancer]		%[No. of Cancer cases/No. of Biopsy specimens]	%[No. of high score cases/No. of Prostatic Cancer Cases]
2007	161	6,7	7.45 (12/161)	50.00 (6/12)
2008	173	6,7	9.24 (16/173)	50.00(8/16)
2009	165	7,6	8.48(14/165)	21.42 (3/14)
2010	208	7,6	10.57 (22/208)	45.45(10/22)
2011	258	7,6	6.58 (17/258)	62.50 (10/17)
2012	246	7,6	11.78 (29/246)	48.27(14/29)
2013	257	6,7	10.89(28/257)	39.28 (11/28)
2014	261	7,6	12.26(32/261)	59.37 (19/32)
2015	353	6,7	11.89 (42/353)	54.76 (23/42)
2016	304	6,7	14.47(44/304)	50.00(22/44)
Gross Mean	NA	68.51±9.22(SD)	10.72(256/2386)	NA
5 year Interval Mean difference[(2007-2011)% -(2012-2016)%]	NA	NA	3.92(~4)	NA

## Discussion

Our retrospective investigation reveals that almost 10.7% Pakistani patients became subject to an incidental finding of prostatic cancer among their TURP specimens. This is a high percentage when compared with various Asiatic and non-Asiatic population as illustrated in Table 2 [13,16-27]. Before addressing the high burden of incidental prostate cancer and its possible progression to aggressive metastasis, some logical explanations must be sought to justify this high digit.

**Table 2:** comparison of incidental prostatic cancer with Asiatic and non-Asiatic populations

Year/Duration of Study	Authors , Country of Origin	Prostate Cancer Incidence %
	**[Asiatic Populations]**	[No. of Prostate Cancer cases/No. of Biopsy specimens] %
**2007-2017**	Janjua TK *et al*. Pakistan	10.7(256/2387)
**2010-2015**	Varghese J *et al*. India [16]	5.2 (31/597)
**2004-2008**	Yoo C *et al*. Korea [17]	4.8 (78/1613)
**2004-2014**	Lin J *et al*. China [13]	8.6 (87/1024)
	**[Non-Asiatic Populations]**	
**2004-2008**	Voight S *et al*. Germany [18]	11.1 (111/1000)
**2005-2007**	Bright EA *et al*. Leicester, United Kingdom [19]	11.4 (47/411)
**1994-2000**	Jones J *et al*. Cleveland, United States [20]	5.2 (26/501)
**1996-2006**	Biers SM *et al*. Winchester, United Kingdom [21]	4.1 (23/680)
**2006-2007**	Trpkov K *et al*. Alberta, Canada [22]	16.7 (126/747)
**2006**	Antunes AA *et al*. Sao Paulo, Brazil [23]	1.8 (3/168)
**1979-1998**	Di silvero F *et al*. Rome, Italy [24]	5.5 (217/3942)
**1993**	Zigeuner RE *et al*. Austria [25]	13 (314/2422)
**1992-1999**	Merrill RM *et al*. Utah, United States [26]	10.5 (675/6426)
**1997-1999**	Mai KT *et al*. Ontario, Canada [27]	8.0 (36/449)

Pakistan´s population has vastly increased over the last decades and as of now is the world´s fifth most populous country [28] and fourth in Asia. Increasing number of elderly males can explain the increased overall incidence and 5 year interval rise. Secondly owing to improved medication availability, procurement and better health coverage over the years the average life span of the elderly male has also increased. Our results and past established data prove that prostate cancer maladies are positively co-related with increasing age [29] and later decades in life. Thus, a higher life expectancy can account for greater cases of BPH and resulting incidental cancers in the prostate gland. This is evident from literature published by the western developed nations like Germany [18], United Kingdom [19], Canada [22], Austria [25] and the United States [26] whose male elderly populations enjoy relatively higher life expectancies and regular Prostate Specific Antigen (PSA) level screenings. However differences are also evident within the same population pool as depicted in Table 2 [13,16-27] among American, British and Canadian populations. Ethnicity, lifestyle and genetic variations could account for these geographic differences and are worthy of additional investigations so that different ethnicities within the same population pool can be identified with specific risks for developing prostate cancer.

Keeping in mind above discussed variations we must give special attention to the majority proportion of higher Gleason score prostatic adenocarcinoma patients. This further warrants greater investigation among the Pakistani populations which are an ethnically diverse group of people. It will certainly aid in understanding and establishing a uniform health care system which regularly screens patients through measuring PSA levels for addressing the increasing burden of prostate cancer. Pakistan already displays a high general prostate cancer incidence [4] yet necessary regular screening of PSA levels at old age is scarce. It is logical to assume that prior education and management of Prostatic Cancer will certainly assist in negating the unwanted lifestyle and mental health changes that a malignancy can bring particularly during old age. That being said; prospective studies need to be conducted on patients with incidental cancer findings for determining the behaviour and outcome of their tumors.

It is imperative to mention that Pakistan despite harbouring one of the highest incidences of cancer including but not limited to Breast, Oral and Prostate Cancers lacks integrated national tumor registries. Burghuri *et al*. [4] established the blueprints for a sustainable national tumor registry system in the year 2000. Electronic medical record (EMR) systems and specific Cancer registries must be nationally established and regularly updated so changes and possible rise in cancer incidences can be timely documented and investigated.

**Limitations of the study**: the study involved inclusion of only TURP specimens and it accounts for all incidental Prostate Cancers, the majority of which included adenocarcinomas. Observational remarks from the study: Pakistan owing to its poor economic infrastructure and low literacy rate suffers from poor health education. It was observed that some patients travelling from low lying areas and provinces of Balochistan, KPK and Sindh often do not reach back for additional reports and follow up unless serious symptoms provoke them to. It is beyond the scope of this study to investigate this but we feel it merits mention for addressing the education of such patients as well.

## Conclusion

The high incidental prostatic cancer figure requires investigation for a developing nation like Pakistan. An update and digital overhaul for informative Cancer registry portals must be acknowledged. Furthermore similar investigations among different provinces must be conducted to investigate and understand any differences owed to ethnicity, lifestyle and genetic variations. We believe our 10 year retrospective analysis is useful for Asiatic and non-Asiatic populations willing to address the burden of prostatic cancers worldwide. Strengths of the study: our study includes a vast patient pool ranging 10 years involving 2386 TURP specimens. Our hospital´s Histopathology department located centrally in the city of Karachi receives pathology specimens from all over Pakistan, hence drawing a central picture regarding Prostate Gland diseases. We feel our study presents the largest data so far studied in Pakistan.

### What is known about this topic

- Incidental prostatic cancer findings have modestly been reported and discussed for different populations before;
- Differences among various populations have been documented before.

### What this study adds

- Our study documents findings; to the best of our knowledge the largest sample size over a 10 year period with diverse patients reporting from all over Pakistan & a comprehensive comparison with similar major findings;
- Our findings support and underscore the rising trend of increasing incidence over the last decade in an underdeveloped nation;
- Our findings further highlight majority proportion of incidental prostatic tumors having higher Gleason scores.
